# Verifications

**Published:** 1890-05

**Authors:** E. T. Balch

**Affiliations:** South Bend, Washington


### VERIFICATIONS.

E. T. Balch, M. D., South Bend, Washington.

July 9th, 1889.—M., infant, one month. Spotted in a most interesting manner on scalp, face, and chest with ringworm (tinea tricophytina). Face pale, body cold, nurses fairly well, digestion good, sleeps mostly at night. Parents greatly annoyed; fear that child will be disfigured. Sepia,$^6$ one dose, dry on tongue. The mother being a blonde, decided to administer to the child direct.

July 10th.—Sac.-lac. three times a day in water.

July 14th.—Ringworms, dusky looking, many. Can be rubbed off. Continue S. L.

July 25th.—Child's skin clear; only a few spots can be detected on chest. Continue treatment.

July 30th.—Perfectly well. Skin normal; not a vestige of ringworm to be discovered.

Dismissed the case—cured.

---

Miss Kate, aged eleven, brunette, tall, angular, with hectic flush, had been coughing some few days which finally culminated in croupous pneumonia with the characteristic dusky face, hurried respiration, and rusty-colored sputum. Temp. 103–104½°. Resp. 30–35. Pulse 125.40. Verat.-vir., Phos., and Bry. were each prescribed as indicated, but with negative results. The fever would not down, but like Banquo's ghost, was ever present to alarm and terrify the parents and endanger the life of the patient. Finally, on the ninth day, fearing the worst, I was urged by the parents to stay if only for one night, and as all the family were fatigued and worn out by long watching, I begged them to retire and took charge of the case myself. The outlook was serious enough, but toward midnight the patient became very restless, and, feeble as she was, I noticed she endeavored to uncover herself—would not tolerate clothing. This was plainly enough Sulph. So at midnight I gave Sulph.[5], a dose. In forty-five minutes sweat appeared on the face. Temperature fell, respiration diminished, and by daylight when I called the parents I gave them joy by pronouncing her better, in fact, out of danger. Did the Sulph. arouse the dormant absorbents, and thereby enable the previous prescription to act?

